# Associations between abdominal obesity and the prevalence of fractures among Chinese adults: insights from a nationwide cross-sectional study

**DOI:** 10.3389/fendo.2026.1759573

**Published:** 2026-02-25

**Authors:** Wenting Qi, Wei Yu, Qianqian Pang, Lin Chen, Shunyu Tang, Hua Lin, Lu Cui, Xiaolan Jin, Zhongjian Xie, Zhixin Li, Mei Li, Yan Jiang, Linhong Wang, Xiangjun Yin, Lijia Cui, Weibo Xia

**Affiliations:** 1 Department of Endocrinology, Key Laboratory of Endocrinology, Peking Union Medical College & Chinese Academy of Medical Sciences, Beijing, China; 2 National Commission of Health, The State Key Laboratory for Complex, Severe, and Rare Diseases, Peking Union Medical College Hospital, Beijing, China; 3 Department of Internal Medicine, Plastic Surgery Hospital, Peking Union Medical College & Chinese Academy of Medical Sciences, Beijing, China; 4 Department of Radiology, Peking Union Medical College Hospital, Peking Union Medical College & Chinese Academy of Medical Sciences, Beijing, China; 5 Department of Wound Repair and Rehabilitation Medicine, State Key Laboratory of Trauma, Burns and Combined Injury, Daping Hospital, Army Medical University, Chongqing, China; 6 Division of Elderly Health, National Center for Chronic and Noncommunicable Disease Control and Prevention, Chinese Center for Disease Control and Prevention, Beijing, China; 7 Department of Orthopedics, Nanjing Drum Tower Hospital, the Affiliated Hospital of Nanjing University Medical School, Nanjing, Jiangsu, China; 8 Department of Endocrinology, Chengdu Military General Hospital, Chengdu, Sichuan, China; 9 Hunan Provincial Key Laboratory of Metabolic Bone Diseases, National Clinical Research Center for Metabolic Diseases, Changsha, Hunan, China; 10 Department of Metabolism and Endocrinology, The Second Xiangya Hospital of Central South University, Changsha, Hunan, China; 11 National Center for Chronic and Non-communicable Disease Control and Prevention, Chinese Center for Disease Control and Prevention, Beijing, China

**Keywords:** abdominal obesity, balance ability, bone mineral density, fractures, osteoporosis

## Abstract

**Purpose:**

To investigate the links between abdominal obesity and skeletal outcomes in a population-based cohort in China.

**Methods:**

8,251 participants from the COPS cohort were enrolled in this study and categorized by quartiles of abdominal obesity indices. Abdominal obesity was determined using sex-specific waist circumference (WC) cutoffs (≥ 90 cm for males, ≥ 85 cm for females), or a waist-to-height ratio (WHtR) ≥0.5. Physical performance and balance were assessed using the Five-Repetition Sit-to-Stand Test (5R-STS) and the Sharpened Romberg test. Vertebral fractures were identified by spine X-ray examination, while information on clinical fractures in recent 5y were collected by a self-report questionnaire. Multivariate regression models were employed with covariate adjustments. Predictive capacity of adiposity metrics for fractures was assessed through ROC analysis.

**Results:**

Abdominal obesity was linked to lower bone turnover rate and higher BMD. However, it was also significantly associated with impaired balance, evidenced by prolonged 5R-STS times and higher rates of positive Sharpened Romberg tests. The prevalence of vertebral fractures and clinical fractures in recent 5y increased across quartiles of WC, WHtR, and Chinese visceral adiposity index (CVAI) (all p< 0.001). An increment of one standard deviation (SD) in these indices was linked to a 19%–30% greater prevalence of fractures. These findings remained robust after limiting analysis to Genant grade ≥2 vertebral fractures. BMI-stratified analysis revealed that abdominal obesity independently increased the prevalence of vertebral fractures regardless of BMI. Notably, normal-weight individuals with abdominal obesity had a 1.75-fold higher prevalence of vertebral fractures (*p* = 0.022), while BMI alone showed no significant effect. Moreover, WHtR demonstrated superior predictive capacity for vertebral fractures and Genant grade ≥2 vertebral fractures vs BMI (AUC 0.62 vs 0.52), with optimal performance when combined with age, sex, fracture history, and BMD (AUC = 0.80).

**Conclusion:**

Abdominal obesity was independently associated with impaired balance and elevated prevalence of vertebral fractures and clinical fractures, even in normal-weight individuals. WHtR demonstrated superior discriminative power for vertebral fractures.

## Introduction

Osteoporotic vertebral fractures impose dual burdens through their epidemic prevalence, which is rising from 15% to 23.9% within a decade (1, 2), and cascading fracture risk in aging population (3). Although obesity has long been regarded as a protective factor for bone mineral density (BMD), emerging evidence indicates that its relationship with fracture risk may be multifaceted and may vary depending on fat accumulation and metabolic health (4). As the most widely used anthropometric measure to define obesity, Body mass index (BMI) fails to distinguish fat mass and lean mass and does not reflect regional fat distribution, especially in aging populations. Age-related sarcopenia and adipose tissue redistribution (5) further reduce the utility of BMI in differentiating metabolically protective lean mass from harmful adiposity. Therefore, alternative adiposity indices that better capture the metabolic implications of obesity are needed.

Nationwide data by the China PEACE Million Persons Project indicate that abdominal obesity affects nearly half (46.3%) of Chinese women aged 65–75 years (6). Emerging evidence highlights abdominal obesity metrics as superior predictors of metabolic derangements compared to BMI (7), with strong associations to insulin resistance, cardiovascular diseases, and diabetes mellitus (8, 9). From a biological perspective, excess visceral fat may adversely affect bone metabolism through chronic low-grade inflammation (10) and may further increase fall risk (11), due to impaired physical function. These findings emphasize the critical importance of further clarifying the skeletal consequences of abdominal adiposity. Practical anthropometric indices, including waist circumference (WC), waist-to-hip ratio (WHR), waist-to-height ratio (WHtR) and novel composite measures like Chinese visceral adiposity index (CVAI) (12), offer cost-effective alternatives to imaging modalities for assessing visceral adiposity. However, current understanding of central obesity-fracture relationships remains controversial. A prospective cohort consistent of over 54,000 female nurses revealed that women with WC ≥108 cm exhibited 1.76 times greater likelihood of vertebral fractures (95% CI 1.06-2.92, p<0.01) compared to those with WC<71cm (13). Parallel findings emerged from Korean nationwide analyses, indicating abdominal obesity thresholds predicted 23-37% higher risks of vertebral fractures in women (14). A Norwegian cohort found abdominal obesity increased hip fracture risk (15), contrasting with European findings showing no significant relationship between WHR and hip fracture risk (16). Meta-analyses further identified that WHtR-defined central obesity was correlated with higher hip fracture risk (17). Collectively, existing evidence points to a potential connection between abdominal obesity and the risk of fractures, yet this association remains insufficiently defined.

Over the past decades, China has experienced a marked escalation in the prevalence of abdominal obesity, driven by rapid industrialization and lifestyle changes (18). Data from the China Health and Nutrition Survey indicated that the prevalence of abdominal obesity among Chinese adults increased from approximately 20% in the early 1990s to over 40% by the 2010s, reflecting a more than two-fold rise within three decades (19). Notable differences exist between China and Western countries in obesity classification criteria, particularly for BMI-defined obesity, as well as in body composition patterns and fracture risk profiles, further influenced by variations in ethnicity, nutritional status, socioeconomic conditions, and cultural factors. For example, Chinese guidelines define general obesity as BMI ≥28.0 kg/m², whereas WHO criteria use BMI ≥30.0 kg/m² (20). The lack of large-scaled data limits our understanding of whether abdominal obesity confers additional fracture risk beyond that predicted by BMI in the Chinese population, particularly in younger populations who may not yet meet traditional criteria for osteoporosis. Additionally, it remains unknown whether different obesity indicators, including BMI, WC, WHtR, and CVAI, exhibit varying abilities to predict fractures.

Therefore, our analysis was designed to investigate the link between abdominal obesity and the prevalence of fractures in a nation-wide cohort of Chinese population, and compare the discriminative power of different obesity indicators for fractures.

## Methods

### Study population

**Figure 1:**
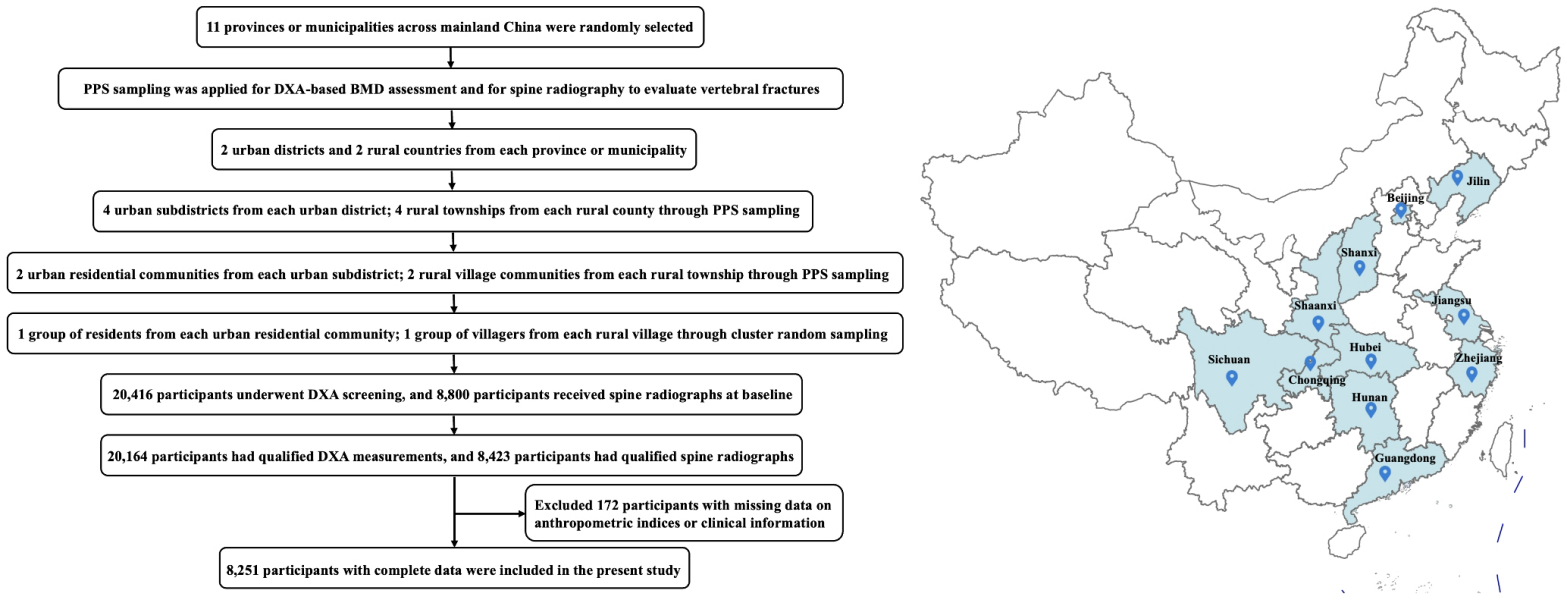
Flowchart of participant selection and geographic distribution of study sites in the COPS cohort.

The China Osteoporosis Prevalence Study (COPS) was a large-scale, nationwide epidemiological survey targeting adults aged ≥20 years, conducted between December 2017 and August 2018 across representative regions of mainland China. To ensure population representativeness, a multistage, stratified, clustered random sampling method was utilized as described previously (21). Briefly, eleven provinces/municipalities were randomly selected, from which two urban districts and two rural counties per site were chosen using probability-proportional-to-size (PPS) sampling, a standard method in multistage population-based surveys to ensure representativeness (22, 23). Within each district or county, four rural townships were additionally selected for DXA assessments and spinal radiography. Within each selected region, two urban residential communities and two rural villages were randomly chosen using PPS sampling method to ensure representative coverage. From these, one cluster of residents in urban sites and one cluster of villagers in rural sites were recruited. Eligible participants were permanent residents within the selected study regions≥6 months in the past year. Participants who were unable to communicate effectively or complete the study questionnaires and physical examinations were excluded. Of the initially 8,800 recruited individuals with radiographs, 377 were excluded due to unqualified radiographic data, 38 due to missing biochemical measurements, and 134 due to incomplete questionnaire information. Finaly, a total of 8,251 eligible participants were ultimately included in the current analysis (**Figure 1**). Ethical approval for the study was obtained from the Institutional Review Board of the Chinese Center for Disease Control and Prevention (Approval No. 201805), and all participants provided written informed consent before enrollment.

### Clinical data collection

Demographics, medical history, lifestyle factors, and fracture records were collected via standardized questionnaires as detailed previously (21). Smoking status, alcohol use, and physical activity were self-reported. Hypertension, diabetes, and osteoarthritis were identified by self-report or current medication use.

Fasting blood samples were processed on-site and analyzed centrally. Biochemical markers, including fasting blood glucose (FBG) and lipid parameters, specifically total cholesterol (TC), triglycerides (TG), high-density lipoprotein cholesterol (HDL-C), and low-density lipoprotein cholesterol (LDL-C), were measured using enzymatic colorimetry (Roche Cobas c501). Bone metabolism markers, including total 25-hydroxyvitamin D (25OHD), C-terminal telopeptide of type I collagen (β-CTX) and procollagen type I N-terminal propeptide (P1NP) were measured by electrochemiluminescence immunoassay, following manufacturer protocols.

### Anthropometric measurements and obesity definitions

Height, weight, and WC were measured by trained staff following standardized procedures. BMI was calculated as weight (kg)/height² (m²), WHtR as WC (cm)/height (cm), and the CVAI was calculated using sex-specific formulas as follows (12):

Males:


$$\begin{array}{c} CVAI=\;-267.93+0.68\times age\;\left(y\right)+0.03\times BMI\;\left(\frac{kg}{m^{2}}\right)+4.00\\ \times WC\;\left(cm\right)+22.00\times Lg\;TG\;\left(\frac{mmol}{L}\right)-16.32\times HDL\;\left(\frac{mmol}{L}\right) \end{array}$$

Females:


$$\begin{array}{c} CVAI=-187.32+1.71\times age\;\left(y\right)+4.32\times BMI\;\left(\frac{kg}{m^{2}}\right)+1.12\\ \times WC\;\left(cm\right)+39.76\times Lg\;TG\;\left(\frac{mmol}{L}\right)-11.66\times HDL\;\left(\frac{mmol}{L}\right) \end{array}$$

According to Chinese National Health Industry Standards (WS/T 428-2013), sex-specific criteria were applied to define abdominal obesity with WC ≥90 cm for males and ≥85 cm for females. WHtR ≥0.5 was also used as an alternative threshold. Four BMI strata were defined by Chinese criteria (24): normal-weight (NW, 18.5-23.9 kg/m²), over-weight (OW, 24-27.9 kg/m²), and obese (OB, ≥28 kg/m²). WC, WHtR, and CVAI were also analyzed by quartiles (Q1-Q4) for comparison.

### Ascertainment of bone mass and fractures

BMD at the lumbar spine (LS, L1-L4), femoral neck (FN), and total hip (TH) was measured using dual energy x-ray absorptiometry (DXA) scanners (Hologic Inc., Waltham, MA, USA or GE Healthcare, Madison, WI, USA), with daily calibration and cross-device standardization. BMD values were standardized across scanners via linear regression models as previously validated (25, 26). Of 20,416 participants, 20,164 (98.8%) had valid BMD data. A subset of 8,423 adults aged ≥40 underwent lateral thoracolumbar X-rays for vertebral fractures detection using the Genant semi-quantitative method (grade≥1), confirmed independently by two radiologists (27). Additionally, clinically diagnosed fractures within the past 5 years were self-reported via standardized questionnaires, regardless of trauma mechanism or skeletal site. Due to the large-scale epidemiological design, detailed classification of injury mechanism or minor fracture sites was unavailable; therefore, these were analyzed as overall clinical fractures.

### Balance assessment

Postural stability was objectively assessed using two validated protocols. The Five-Repetition Sit-to-Stand Test (5R-STS) measured the time taken (in seconds) to complete five consecutive sit-stand cycles from a chair of standardized height (46 cm), without the use of upper limb support. The Sharpened Romberg test evaluated the duration of standing in a tandem stance (up to a maximum of 60 seconds), with age-specific thresholds, which was described in detail previously (21).

### Statistical analysis

Continuous variables were summarized as mean ± SD or median [IQR] or medians with interquartile ranges (IQR), while categorical variables were expressed as counts and percentages. Differences among groups were assessed by ANOVA or Kruskal-Wallis tests for continuous data, and χ² or Fisher’s exact tests for categorical variables. Multivariable linear and logistic regression analyses were performed to assess associations between abdominal obesity indices and BMD, fracture prevalence, and balance ability, with covariates adjustment. Restricted cubic splines (RCS) explored the non-linear trends (plotRCS package). ROC analysis evaluated the predictive performance of obesity indicators for fractures, with AUCs compared using the DeLong test and optimal cutoffs determined by Youden’s index (pROC package). Covariate selection followed established fracture risk frameworks. All data analyses were carried out in R software (v4.1.2), and results with p-values below 0.05 were deemed statistically significant.

## Results

### Baseline characteristics of the study participants

Participants were categorized into quartiles (Q1-Q4) according to their abdominal obesity measures. The baseline demographic and clinical data were outlined in **Table 1**. Significant age differences were noted among the WC, WHtR, and CVAI groups, with older participants observed in the Q4 quartiles (*p<*0.05 for all comparisons). We also noticed differences in sex, long-term use of glucocorticoid, smoking, and alcohol consumption (all *p* < 0.001). Fracture history showed a rising trend from Q1 to Q4 groups in each parameter (*p* < 0.001 for all comparisons). Bone turnover markers β-CTX and P1NP levels, were lowest in the Q4 groups, while highest in the Q1 groups (*p* < 0.05 among groups). Vitamin D deficiency prevalence escalated with adiposity severity across all parameters, paralleled by impaired metabolic profiles.

**Table 1:** The comparisons of demographic characteristics among different abdominal obesity indices quartiles.

Characteristics	WC					WHtR					CVAI				
	Q1 (N=2060)	Q2 (N=1952)	Q3 (N=2178)	Q4 (N=2061)	*P-*value	Q1 (N=2055)	Q2 (N=2066)	Q3 (N=2074)	Q4 (N=2056)	*P-*value	Q1 (N=2062)	Q2 (N=2064)	Q3 (N=2062)	Q4 (N=2063)	*P-*value
**Age (years)**	54.0 [47.0, 64.0]	57.0 [49.0, 64.0]	57.0 [50.0, 65.0]	58.0 [50.0, 66.0]	***<0.001***	54.0 [47.0, 63.0]	55.0 [49.0, 64.0]	57.0 [50.0, 65.0]	60.0 [52.0, 67.0]	***<0.001***	53.0 [46.0, 62.0]	55.0 [49.0, 64.0]	58.0 [51.0, 66.0]	61.0 [52.0, 67.0]	***<0.001***
**Sex (female, %)**	1416 (68.74%)	1219 (62.45%)	1224 (56.20%)	880 (42.70%)	***<0.001***	1108 (53.92%)	1123 (54.36%)	1171 (56.46%)	1337 (65.03%)	***<0.001***	1412 (68.48%)	1302 (63.08%)	1146 (55.58%)	879 (42.61)	***<0.001***
**Height (cm)**	157 [152, 162]	158 [153, 164]	160 [154, 166]	163 [156, 169]	***<0.001***	161 [156, 167]	160 [155, 166]	159 [154, 165]	156 [151, 163]	***<0.001***	158 [153, 162]	158 [153, 164]	160 [154, 166]	163 [156, 168]	***<0.001***
**Weight (kg)**	52.8 [48.3, 56.7]	58.8 [54.6, 63.0]	64.3 [59.6, 69.0]	73.3 [67.1, 79.6]	***<0.001***	55.0 [50.2, 60.5]	60.1 [54.6, 66.3]	64.0 [57.5, 71.1]	68.2 [61.5, 76.5]	***<0.001***	53.1 [48.8, 57.2]	59.3 [54.8, 63.6]	64.3 [59.2, 69.5]	72.7 [66.3, 79.3]	***<0.001***
**BMI (kg/m^2^)**	21.2 [19.8, 22.6]	23.4 [22.0, 24.7]	25.0 [23.7, 26.5]	27.6 [26.1, 29.5]	***<0.001***	21.1 [19.8, 22.5]	23.5 [22.2, 24.7]	25.1 [23.7, 26.6]	27.6 [26.0, 29.7]	***<0.001***	21.3 [19.9, 22.7]	23.4 [22.0, 24.9]	25.0 [23.6, 26.5]	27.5 [25.9, 29.5]	***<0.001***
**Urbanity (n, %)**	1131 (54.90%)	1063 (54.46%)	1209 (55.51%)	1118 (54.25%)	***<0.001***	1158 (56.35%)	1162 (56.24%)	1122 (54.10%)	1079 (52.48%)	***0.035***	1103 (53.49%)	1115 (54.02%)	1158 (56.16%)	1145 (55.50%)	0.275
**Long-term use of glucocorticoids (n, %)**	16 (0.78%)	31 (1.59%)	32 (1.47%)	48 (2.33%)	***0.001***	15 (0.73%)	34 (1.65%)	26 (1.25%)	52 (2.53%)	***<0.001***	15 (0.73%)	35 (1.70%)	31 (1.50%)	46 (2.23%)	***0.001***
**Smoking habits (n, %)**					***<0.001***					***<0.001***					***<0.001***
Current smoking	379 (18.44%)	388 (19.83%)	485 (22.25%)	545 (26.5%)		522 (25.41%)	492 (23.8%)	439 (21.14%)	344 (16.7%)		398 (19.26%)	377 (18.26%)	470 (22.8%)	552 (26.7%)	
Stopped smoking	81 (3.93%)	97 (4.97%)	134 (6.15%)	211 (10.2%)		119 (5.79%)	124 (6.00%)	136 (6.56%)	144 (7.00%)		73 (3.54%)	106 (5.14%)	130 (6.30%)	214 (10.4%)	
Never smoking	1600 (77.7%)	1467 (75.2%)	1559 (71.6%)	1305 (63.3%)		1414 (68.8%)	1450 (70.2%)	1499 (72.3%)	1568 (76.3%)		1591 (77.2%)	1581 (76.6%)	1462 (70.9%)	1297 (62.9%)	
**Drinking habits (n, %)**										***0.017***					***<0.001***
Never drinking	1542 (74.9%)	1370 (70.2%)	1483 (68.1%)	1279 (62.1%)	***<0.001***	1416 (68.9%)	1375 (66.6%)	1413 (68.1%)	1470 (71.5%)		1511 (73.3%)	1447 (70.1%)	1420 (68.9%)	1296 (62.8%)	
Sometimes drinking	339 (16.5%)	380 (19.5%)	443 (20.3%)	497 (24.1%)		396 (19.3%)	449 (21.7%)	428 (20.6%)	386 (18.8%)		349 (16.9%)	405 (19.6%)	421 (20.4%)	484 (23.5%)	
Drinking less than 6 units/per week	143 (6.94%)	164 (8.40%)	205 (9.41%)	211 (10.2%)		189 (9.20%)	194 (9.39%)	193 (9.31%)	147 (7.15%)		158 (7.66%)	181 (8.77%)	169 (8.20%)	215 (10.4%)	
Drinking over 6 units/per week	36 (1.75%)	38 (1.95%)	47 (2.16%)	74 (3.59%)		54 (2.63%)	48 (2.32%)	40 (1.93%)	53 (2.58%)		44 (2.13%)	31 (1.50%)	52 (2.52%)	68 (3.30%)	
**History of ever fractures (n, %)**	240 (11.7%)	282 (14.4%)	316 (14.5%)	332 (16.1%)	***0.002***	237 (11.5%)	308 (14.9%)	299 (14.4%)	326 (15.9%)	***0.003***	236 (11.4%)	298 (14.4%)	306 (14.8%)	330 (16.0%)	***0.002***
**FBG (mmol/L)**	5.46±1.32	5.68±1.55	5.91±1.73	6.19±1.92	***<0.001***	5.48±1.50	5.70±1.43	5.91±1.75	6.17±1.88	***<0.001***	5.37±1.10	5.71±1.65	5.91±1.72	6.27±1.96	***<0.001***
**TC (mmol/L)**	4.86±0.93	4.94±1.02	4.97±1.01	5.00±1.02	***<0.001***	4.79±0.98	4.97±0.99	4.98±1.00	5.07±1.02	***<0.001***	4.85±0.91	4.92±0.97	5.00±1.04	5.01±1.07	***<0.001***
**TG (mmol/L)**	1.45±0.90	1.49 [1.13, 2.12]	1.67 [1.24, 2.38]	1.95 [1.42, 2.76]	***<0.001***	1.22 [0.96, 1.63]	1.51 [1.13, 2.10]	1.73 [1.26, 2.44]	1.90 [1.41, 2.67]	***<0.001***	1.15 [0.94, 1.48]	1.46 [1.13, 1.98]	1.74 [1.29, 2.45]	2.14 [1.54, 3.06]	***<0.001***
**HDL-C (mmol/L)**	1.63±0.44	1.44±0.39	1.35±0.37	1.26±0.35	***<0.001***	1.559±0.45	1.44±0.40	1.33±0.38	1.31±0.36	***<0.001***	1.71±0.44	1.46±0.36	1.31±0.33	1.19±0.32	***<0.001***
**LDL-C (mmol/L)**	2.95±0.85	2.99±0.90	3.02±0.94	2.98±0.95	***0.024***	2.89±0.85	3.00±0.90	3.02±0.94	3.03±0.95	***<0.001***	2.94±0.83	3.05±0.90	3.05±0.94	2.91±0.98	***<0.001***
**β-CTX (ng/dL)**	0.30 [0.21, 0.43]	0.30 [0.21, 0.42]	0.30 [0.21, 0.41]	0.29 [0.21, 0.40]	***0.017***	0.30 [0.20, 0.42]	0.29 [0.21, 0.41]	0.30 [0.22, 0.42]	0.30 [0.21, 0.41]	0.53	0.30 [0.20, 0.42]	0.30 [0.21, 0.41]	0.30 [0.22, 0.42]	0.29 [0.20, 0.40]	***0.031***
**P1NP (ng/dL)**	52.7 [39.4, 69.6]	52.3 [39.4, 68.4]	51.0 [38.7, 66.5]	49.2 [37.3, 65.0]	***<0.001***	50.9 [38.5, 67.2]	50.4 [37.8, 66.8]	51.1 [38.9, 66.3]	52.3 [39.6, 69.1]	0.10	52.1 [38.9, 69.4]	51.9 [39.2, 68.2]	51.9 [39.4, 67.2]	49.2 [37.1, 64.7]	***<0.001***
**25OHD (ng/dL)**	25.0 [19.1, 32.8]	25.3 [19.4, 32.3]	24.4 [18.8, 31.8]	24.3 [19.0, 31.4]	***0.024***	25.6 [19.2, 33.5]	25.5 [19.4, 32.9]	24.6 [19.1, 31.8]	23.4 [18.6, 29.9]	***<0.001***	25.1 [19.1, 32.9]	25.4 [19.5, 32.4]	24.4 [18.8, 31.4]	24.1 [18.8, 31.3]	***0.001***

^1^ Data presented as mean±SD or median [interquartile range] or percentages% (n, %).

^2^ Abbreviation: WC, waist circumference; WHtR, waist to height ratio; CVAI, Chinese visceral adiposity index; BMI, body mass index; FBG, fasting blood glucose; TC, total cholesterol; TG, total triglyceride; HDL-C, high density lipoprotein cholesterol; LDL-C, low density lipoprotein cholesterol; β-CTX, C-terminal cross-linking telopeptide of type I collagen; P1NP, procollagen type I N-terminal propeptide; 25OHD, total 25 hydroxyvitamin D.

^3^ Significant values (p<0.05) are presented in bold italics.

### Association between abdominal obesity and BMD

**Figure 2:**
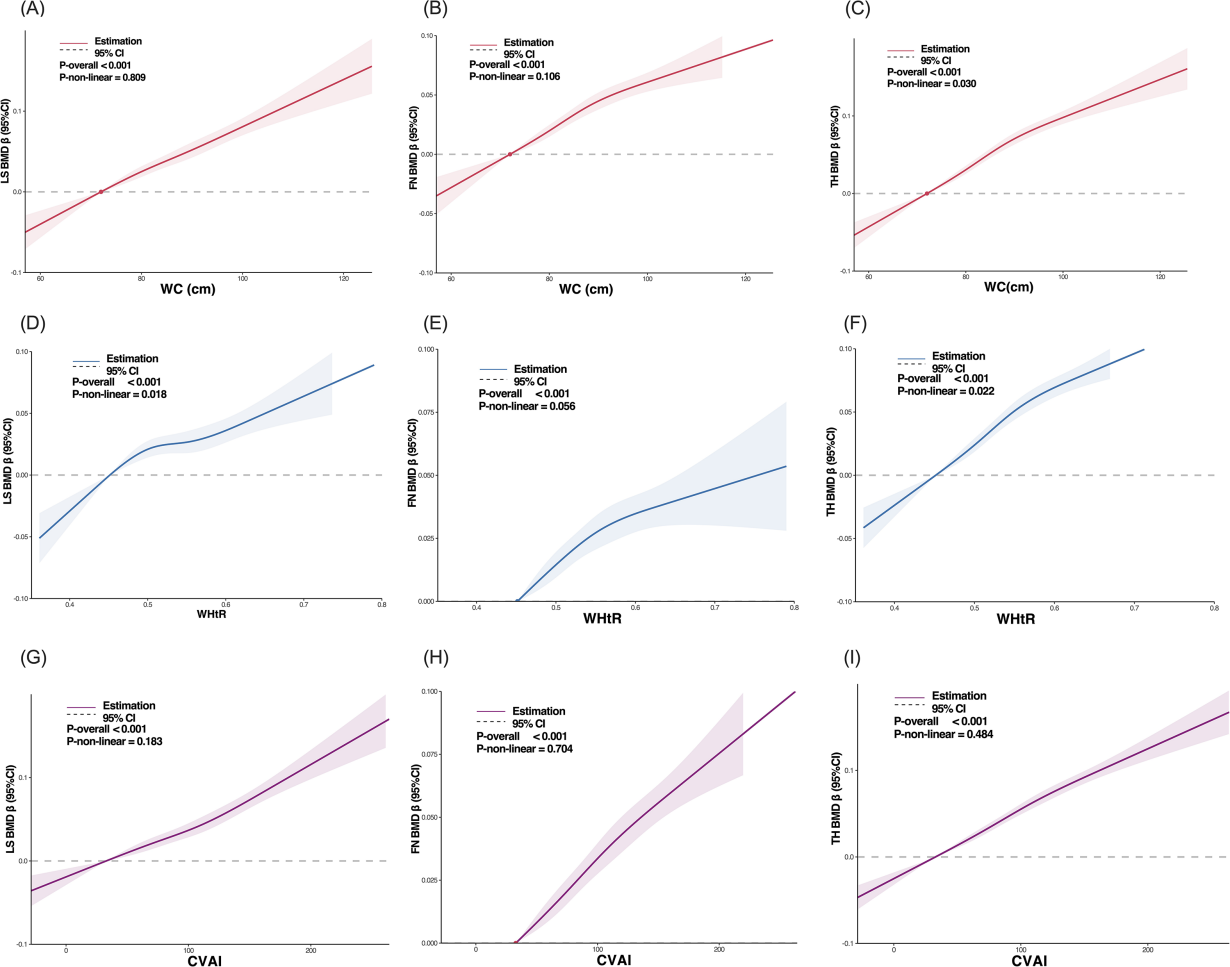
RCS analysis for the associations between WC **(A-C)**, WHtR **(D-F)**, CVAI **(G-I)** and BMD at the lumbar spine, femoral neck, and total hip. Models were adjusted for age, sex, urban-rural difference, smoking and drinking status, diabetes mellitus, hypertension, osteoarthritis, daily carbonated beverage, daily tea, daily coffee, and daily exercise duration. Y-axis ranges differ across panels due to automatic scaling. Shaded areas represent 95% CI.

**Table 2** demonstrated that BMD at the LS, FN, and TH exhibited progressive increased across ascending quartiles of abdominal obesity parameters. The Q1 groups showed the lowest BMD values, whereas the Q4 groups demonstrated significantly elevated BMD at all three skeletal sites, with these differences remaining statistically significant after adjustment for age, sex, urban-rural difference, smoking and drinking status, diabetes mellitus, hypertension, osteoarthritis, daily carbonated beverage, daily tea, daily coffee, and daily exercise duration (multivariable-adjusted *p<*0.001 for all comparisons). As shown in **Figure 2**, RCS analysis confirmed approximately linear positive associations between WC or CVAI and BMD (*p*-nonlinear >0.05 for all sites). A nonlinear association between WHtR and BMD at the LS (*p*-nonlinear=0.018) and TH (*p*-nonlinear=0.022), characterized by attenuated slope gradients at extreme WHtR levels.

**Table 2:** The comparisons of BMD across quartiles of abdominal obesity indices with multiple adjustments.

Characteristics	LS BMD (g/cm^2^)	*p-*value	FN BMD (g/cm^2^)	*P-*value	TH BMD (g/cm^2^)	*P-*value
WC						
Q1 (Ref)	0.89 [0.77, 1.02]	/	0.71 [0.62, 0.80]	/	0.78 [0.68, 0.88]	/
Q2	0.90 [0.78, 1.03]	***<0.001***	0.73 [0.64, 0.83]	***<0.001***	0.82 [0.72, 0.91]	***0.008***
Q3	0.92 [0.82, 1.05]	***<0.001***	0.75 [0.65, 0.84]	***<0.001***	0.84 [0.74, 0.93]	***<0.001***
Q4	0.96 [0.85, 1.08]	***<0.001***	0.77 [0.68, 0.86]	***<0.001***	0.88 [0.78, 0.98]	***<0.001***
WHtR						
Q1 (Ref)	0.91 [0.80, 1.04]	/	0.74 [0.65, 0.83]	/	0.81 [0.71, 0.90]	/
Q2	0.93 [0.81, 1.05]	***<0.001***	0.74 [0.65, 0.84]	***<0.001***	0.83 [0.73, 0.92]	***<0.001***
Q3	0.92 [0.81, 1.05]	***<0.001***	0.75 [0.65, 0.84]	***<0.001***	0.84 [0.73, 0.94]	***<0.001***
Q4	0.92 [0.80, 1.04]	***<0.001***	0.73 [0.65, 0.83]	***<0.001***	0.84 [0.74, 0.95]	***<0.001***
CVAI						
Q1 (Ref)	0.90 [0.78, 1.03]	/	0.72 [0.63, 0.82]	/	0.80 [0.69, 0.89]	/
Q2	0.91 [0.79, 1.04]	***<0.001***	0.74 [0.64, 0.83]	***<0.001***	0.82 [0.72, 0.92]	***<0.001***
Q3	0.92 [0.81, 1.04]	***<0.001***	0.74 [0.65, 0.84]	***<0.001***	0.83 [0.73, 0.93]	***<0.001***
Q4	0.95 [0.84, 1.08]	***<0.001***	0.76 [0.67, 0.86]	***<0.001***	0.87 [0.77, 0.97]	***<0.001***

^1^ Data presented as median [interquartile range]; quartiles (Q1-Q4) are based on the distribution of each abdominal obesity index (WC, WHtR, and CVAI) and Q1 serves as the reference group.

^2^ WC, waist circumference; WHtR, waist to height ratio; CVAI, Chinese visceral adiposity index; BMD, bone mineral density; LS, lumbar spine; FN, femoral neck; TH, total hip.

^3^ Significant values (p<0.05) are presented in bold italics.

### Associations between abdominal obesity and balance ability

The links between abdominal obesity parameters and balance, after controlling for covariates, were detailed in **Table 3**. Participants with higher WC, WHtR, and CVAI values demonstrated longer 5R-STS durations and increased likelihood of a positive Sharpened Romberg test. Compared to Q1, WC Q3 and Q4 were associated with 32% and 78% increases in 5R-STS time, respectively (*p* < 0.01), and elevated Sharpened Romberg test prevalence (OR 1.26 and 1.32, respectively, both *p* < 0.05). Each SD increase in WC corresponded to a 27% rise in 5R-STS time and a 10% higher Sharpened Romberg test prevalence (*p* = 0.0045). WHtR Q4 showed a 30% increase in 5R-STS time and a 46% higher prevalence of positive Sharpened Romberg. Each SD increase raised the 5R-STS time by 15% and the prevalence of Sharpened Romberg by 17% (both *p* < 0.001). Participants in the highest CVAI quartile (Q4) exhibited a 72% longer 5R-STS duration and 1.3-fold higher positive Sharpened Romberg test compared with Q1. Per SD increase, 5R-STS time rose by 28% and the prevalence of Sharpened Romberg by 10% (all *p* < 0.01).

**Table 3:** The association between abdominal obesity and the balance ability.

Characteristics	Time for the five-repetition sit-to-stand test		The Sharpened Romberg test	
	β (95%CI)	P value	OR (95%CI)	P value
WC				
Q1	Ref	/	Ref	/
Q2	0.18 [-0.055, 0.41]	0.13	1.17 [0.97, 1.41]	0.11
Q3	0.32 [0.093, 0.55]	***0.0059***	1.26 [1.05, 1.51]	***0.013***
Q4	0.78 [0.54, 1.02]	***<0.001***	1.32 [1.09, 1.59]	***0.0042***
Increase per SD	0.27 [0.19, 0.36]	***<0.001***	1.10 [1.03, 1.17]	***0.0045***
WHtR				
Q1	Ref	/	Ref	/
Q2	-0.030 [-0.26, 0.20]	0.80	1.08 [0.89, 1.32]	0.42
Q3	0.23 [-0.0023, 0.46]	0.052	1.20 [0.99, 1.45]	0.063
Q4	0.30 [0.060, 0.53]	***0.014***	1.46 [1.22, 1.76]	***<0.001***
Increase per SD	0.15 [0.070, 0.24]	***<0.001***	1.17 [1.09, 1.24]	***<0.001***
CVAI				
Q1	Ref	/	Ref	***/***
Q2	0.24 [0.0075, 0.47]	***0.043***	1.11 [0.92, 1.34]	0.28
Q3	0.23 [-0.0073, 0.46]	0.058	1.09 [0.91, 1.32]	0.35
Q4	0.72 [0.48, 0.96]	***<0.001***	1.30 [1.08, 1.57]	***0.0059***
Increase per SD	0.28 [0.19, 0.36]	***<0.001***	1.10 [1.03, 1.18]	***0.0050***

^1^ Multivariate logistic regression was used to adjust covariates for the comparisons of the Sharpened Romberg test among groups, and multi-linear regression was used to adjust covariates for the comparisons of the time for the five-repetition sit-to-stand test among groups.

^2^ Adjusted for age, sex, urban-rural difference, smoking and drinking status, diabetes mellitus, hypertension, osteoarthritis, daily carbonated beverage, daily tea, daily coffee, daily exercise duration, history of fracture, and BMD at the lumbar spine.

^3^ WC, waist circumference; WHtR, waist to height ratio; CVAI, Chinese visceral adiposity index; OR, odds ratio; 95%CI, 95% confidence interval; SD, standard deviation.

^4^ Significant values (p<0.05) are presented in bold italics.

### Associations between abdominal obesity and the prevalence of fractures

As shown in **Table 4**, a consistent positive relationship was observed between all abdominal adiposity metrics and fracture prevalence. Remarkably, vertebral fracture prevalence increased progressively across WC quartiles, independent of covariates (with OR and 95% CI of 1.41[1.12, 1.77] for Q3 and 1.71[1.35, 2.17] for Q4, respectively), with similar patterns observed for WHtR and CVAI (*p<*0.01 for both comparisons). Continuous analyses revealed 19-26% higher prevalence of vertebral fractures per SD increase in abdominal obesity indices (*p<*0.001 for all comparisons). For the prevalence of recent clinical fractures, Q3 and Q4 WC groups showed 58-68% elevated fracture prevalence (with OR and 95% CI of 1.58[1.13, 2.23] and 1.68[1.19, 2.38], *p<*0.01 for both comparisons), while WHtR or CVAI Q4 demonstrated 73-84% increase in the fracture prevalence, respectively. Sensitivity analyses restricted to vertebral fractures of grade 2 or above yielded the strong links between abdominal obesity and the prevalence of fractures. Each one SD increment in these indices corresponded to an approximately 26%-39% elevation in fracture prevalence.

**Table 4:** Multivariate logistic regression for the associations between abdominal obesity indices with the prevalence of fractures.

Characteristics	Vertebral fractures		Vertebral fractures≥grade2			Clinical fractures in in recent 5y	
	OR (95%CI)	*P*-value	OR (95%CI)		*P*-value	OR (95%CI)	*P*-value
WC							
Q1	Ref	/	Ref		/	Ref	/
Q2	1.36 [1.07, 1.73]	***0.011***	1.08 [0.75, 1.57]		0.67	1.18 [0.81, 1.70]	0.39
Q3	1.44 [1.14, 1.81]	***0.0023***	1.40 [0.99, 1.98]		0.060	1.58 [1.13, 2.23]	***0.0084***
Q4	1.76 [1.39, 2.21]	***<0.001***	1.99 [1.42, 2.81]		***<0.001***	1.68 [1.19, 2.38]	***0.0034***
Increase per SD	1.22 [1.13, 1.32]	***<0.001***	1.30 [1.15, 1.46]		***<0.001***	1.19 [1.06, 1.34]	***0.0026***
WHtR							
Q1	Ref	/	Ref		/	Ref	/
Q2	1.14 [0.89, 1.45]	0.29	1.40 [0.94, 2.11]		0.098	1.15 [0.80, 1.65]	0.45
Q3	1.26 [0.99, 1.59]	0.061	1.46 [0.99, 2.19]		0.059	1.39 [0.98, 1.96]	0.065
Q4	1.88 [1.50, 2.37]	***<0.001***	2.66 [1.86, 3.88]		***<0.001***	1.82 [1.31, 2.55]	***<0.001***
Increase per SD	1.26 [1.16, 1.36]	***<0.001***	1.39 [1.24, 1.55]		***<0.001***	1.17 [1.05, 1.31]	***0.0056***
CVAI							
Q1	Ref	/	Ref		/	Ref	/
Q2	1.27 [0.99, 1.62]	0.057	1.08 [0.74, 1.58]		0.68	1.21 [0.85, 1.74]	0.29
Q3	1.43 [1.13, 1.81]	***0.0033***	1.47 [1.04, 2.12]		***0.033***	1.48 [1.04, 2.10]	***0.028***
Q4	1.61 [1.28, 2.04]	***<0.001***	1.68 [1.18, 2.41]		***0.0044***	1.73 [1.22, 2.45]	***0.0020***
Increase per SD	1.19 [1.10, 1.29]	***<0.001***	1.26 [1.12, 1.43]		***<0.001***	1.22 [1.08, 1.37]	***0.0011***

^1^ Multivariate logistic regression was used to adjust covariates compared with the Q1 group.

^2^ Adjusted for age, sex, urban-rural difference, smoking and drinking status, diabetes mellitus, hypertension, osteoarthritis, daily carbonated beverage, daily tea, daily coffee, daily exercise duration, history of fracture, the Sharpened Romberg test, and BMD at the lumbar spine.

^3^ WC, waist circumference; WHtR, waist to height ratio; CVAI, Chinese visceral adiposity index; OR, odds ratio; 95%CI, 95% confidence interval; SD, standard deviation.

^4^ Significant values (p<0.05) are presented in bold italics.

### Association between abdominal obesity and prevalence of fractures stratified by BMI

**Figure 3:**
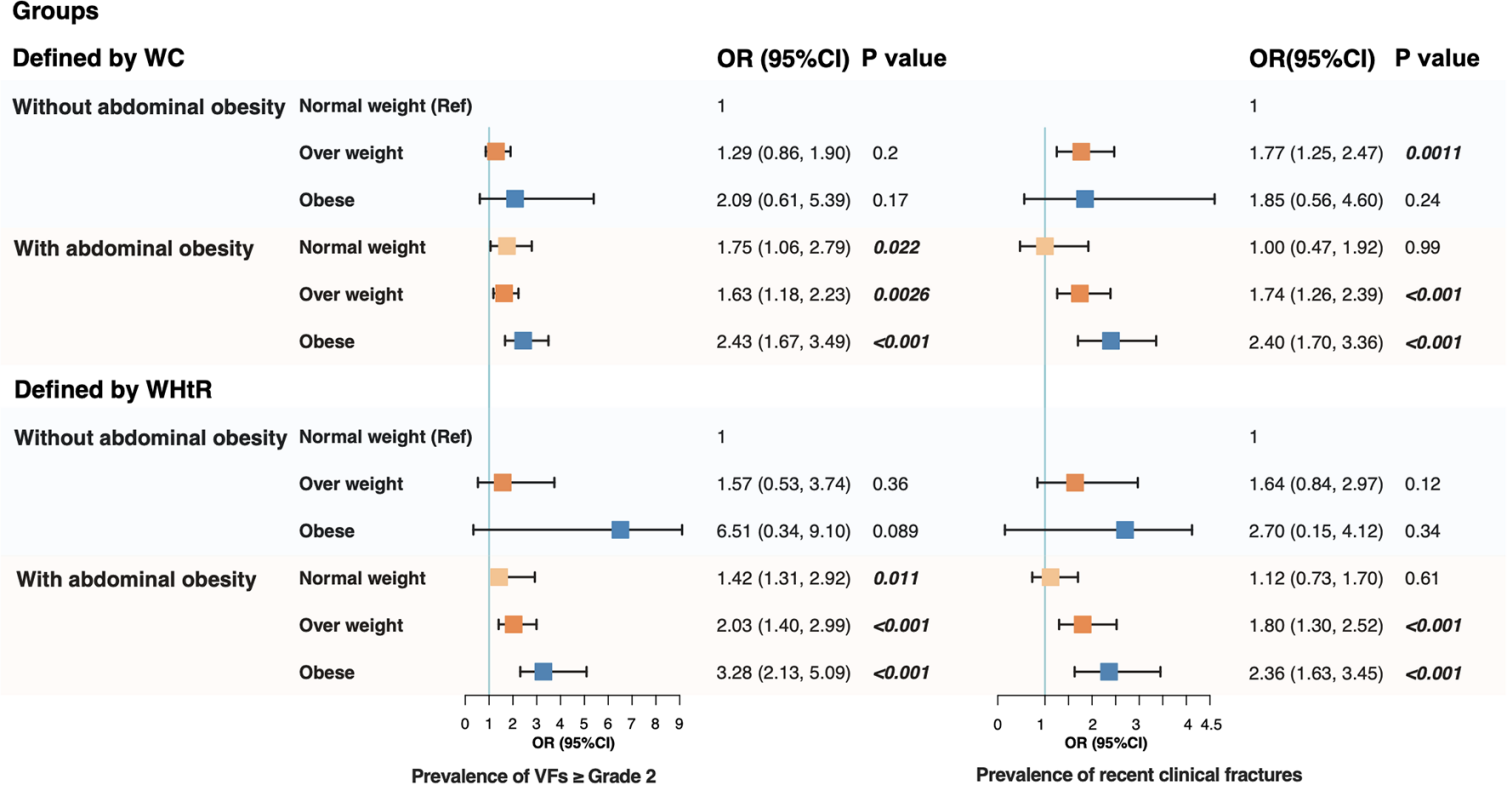
Forest plot for the association between abdominal obesity and fracture prevalence across BMI categories (normal weight, overweight, and obesity) by multivariable logistic regression. The model was adjusted for age, sex, urban-rural difference, smoking and drinking status, diabetes mellitus, hypertension, osteoarthritis, daily carbonated beverage, daily tea, daily coffee, daily exercise duration, history of fracture, the Sharpened Romberg test, and BMD at the lumbar spine.

Given the elevated fracture incidence in overweight/obesity, further analysis stratifiedparticipants by BMI levels with further subdivision based on abdominal obesity, creating six subgroups to evaluate the independent contribution of abdominal obesity to the prevalence of fractures (**Supplementary Table 1**). Underweight individuals (BMI<18.5 kg/m²) were not analyzed separately due to their distinct fracture risk profile and very small sample size, especially among those with abdominal obesity. Participants in the NW without abdominal obesity group was set as the reference group. After adjusting for age, sex, lifestyle, the Sharpened Romberg test, and LS BMD, the prevalence of grade ≥2vertebral fractures significantly increased in OW and OB individuals only when accompanied by abdominal obesity (*p* < 0.05). Notably, even among individuals with NW, participants with abdominal obesity had a higher occurrence of grade ≥2vertebral fractures (*p* < 0.05). When abdominal obesity was defined by WHtR, both the prevalence of vertebral fractures and recent clinical fractures were significantly elevated in OW and OB participants, but only with the presence of abdominal obesity (**Figure 3**). In contrast, an increase in BMI without concurrent abdominal obesity did not significantly affect the prevalence of fractures.

### Obesity parameters and fracture discrimination

**Figure 4:**
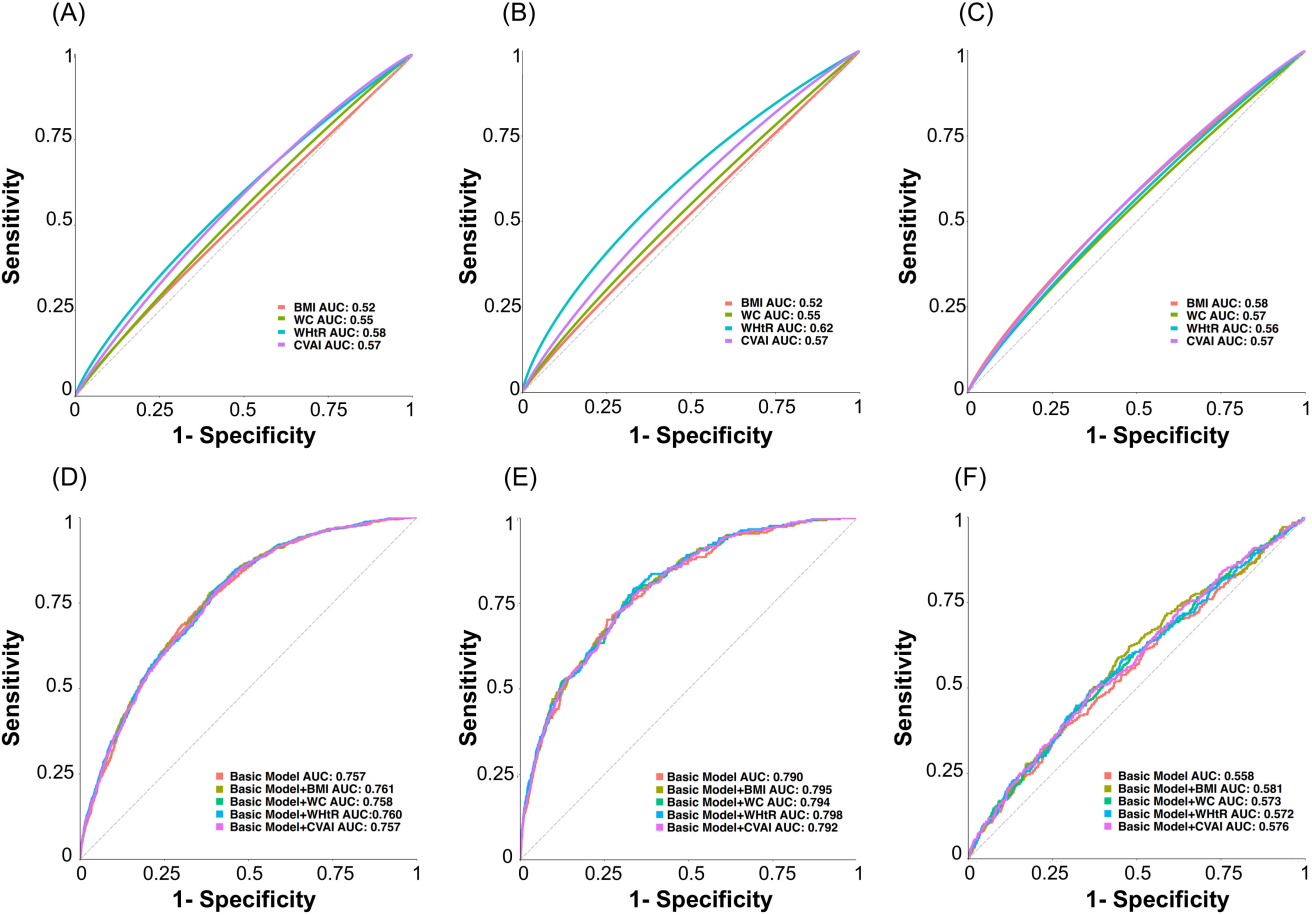
ROC curves comparing the discriminatory performance of different obesity indices alone for predicting **(A)** any-grade vertebral fractures, **(B)** vertebral fractures of grade ≥2, and **(C)** clinical fractures in recent 5y. The incremental predictive performance when these indices are added to a baseline fracture risk model (including age, sex, fracture history, and lumbar spine BMD) for predicting **(D)** any-grade vertebral fractures, **(E)** vertebral fractures of grade ≥2, and **(F)** clinical fractures in recent 5y.

To evaluate the predictive performance differences of various obesity indicators (BMI, WC, WHtR, CVAI) for vertebral fractures and clinical fractures in recent 5y, this study conducted a comparative analysis by plotting ROC curves and calculating the AUC (as shown in **Figure 4**). The results indicated that WHtR demonstrated the optimal discriminatory ability for vertebral fractures (AUC: WHtR 0.58 vs. BMI 0.52, *p<*0.01) and vertebral fractures grade≥ 2 (AUC: WHtR 0.62 vs BMI 0.52, *p<*0.001) with an optimal cut-off value 0.55. Both WC and CVAI also showed significantly better predictive power than BMI (*p<*0.01 for both comparison by Delong test, as shown in **Figures 4A–C**). A basic model incorporating age, sex, fracture history, and LS BMD showed modest vertebral fractures discrimination (AUC = 0.757), slightly improved by BMI inclusion (AUC = 0.761, *p* = 0.04). For vertebral fractures of grade 2 or above, adding WHtR to the basic model significantly improved its predictive performance (AUC 0.80, *p<*0.05 compared with the basic model). However, all models exhibited limited predictive capacity for recent clinical fractures (AUC 0.56-0.58). Although the BMI combination showed a statistical improvement (p=0.027), the magnitude of AUC gain was small, indicating limited incremental predictive value (**Figures 4D–F**).

## Discussion

In this large, cross-sectional, population-based study, we systematically examined the links between abdominal adiposity and skeletal health, including BMD, bone turnover rates, and fracture prevalence among adults in a nationwide Chinese cohort. Abdominal obesity was positively related to BMD and inversely associated with bone remodeling rates. However, abdominal obesity was also linked to impaired balance and increased prevalence of both vertebral and recent clinical fractures. Notably, the combination of WHtR and the Basic Model demonstrated the strongest predictive performance for vertebral fractures, yielding the highest AUC among all evaluated models. Our study primarily evaluated the impact of abdominal obesity on skeletal health in an Asian population including both sexes.

As osteoporosis and abdominal obesity become increasingly prevalent with aging, their coexistence demands closer scrutiny. Although obesity was once thought to protect against bone loss, accumulating evidence, including our findings, suggested a more complex picture: abdominal obesity may be associated with higher BMD, yet paradoxically linked to increased prevalence of fractures. In our study, abdominal obesity was also associated with lower bone turnover markers, indicating a suppressed bone remodeling state. Such low turnover may contribute to relatively higher BMD while potentially impairing bone quality, thereby partly explaining the coexistence of elevated BMD and greater fracture risk. Novel adiposity indices such as WHtR and CVAI, which better capture metabolic disturbances than BMI or WC alone, also appear more informative in assessing skeletal risk. Consistent with data from rural Chinese cohorts, we observed that WHtR was positively correlated with BMD (28). However, unlike NHANES data reporting a U-shaped relationship between visceral adiposity index (VAI) and BMD (29), our results showed no nonlinear association between CVAI and BMD. This discrepancy may reflect population differences, such as lower levels of visceral adiposity in Chinese individuals, or the limited representation of extremely high CVAI values in our cohort (29).

These observations were consistent with previous reports that have documented a direct relationship between abdominal adiposity and a higher likelihood of vertebral fractures, reinforcing the potential detrimental skeletal effects of central fat accumulation. Large-scale studies from South Korean and UK demonstrated increased risk of vertebral fractures with greater visceral fat or WC (14, 30), and a meta-analysis reported a 3% increase in fracture risk for every 10 cm increase in WC (31). A BMI-adjusted positive association between WC and hip fracture risk was also indicated by another meta-analysis (RR = 1.63) (32). Similarly, data from Sweden, Norway, and Canada indicate that elevated WC or WHtR independently predicts higher fracture risk, regardless of BMI status (15, 33, 34). However, not all studies have yielded concordant results. Yamaguchi et al. (35) observed an opposite trend, noting a protective effect of trunk fat against vertebral fractures among type 2 diabetes patients. Likewise, a meta-analysis showed no statistically significant association between abdominal obesity and fracture risk (17). These inconsistencies may stem from population heterogeneity and varying adjustment models. Importantly, our stratified analysis revealed that elevated BMI alone was not associated with greater fracture prevalence unless accompanied by abdominal obesity. The combination of high BMI and central obesity might markedly increase the prevalence of fractures, particularly for grade ≥2 vertebral fractures. This highlights the critical role of abdominal adiposity, rather than general obesity, in fracture risk assessment.

Despite widespread use of BMI in obesity assessment, its limitations in reflecting fat distribution reduce its utility in fracture risk prediction. BMI and waist-based indices reflect different dimensions of adiposity. BMI does not distinguish fat from lean or bone mass, whereas waist-based measures better capture central and visceral adiposity, which is metabolically active and potentially detrimental to bone quality. This study compared the comparative discriminative value of BMI, WC, WHtR, and CVAI in predicting various fracture outcomes, with a focus on vertebral fractures, the most prevalent fragility fracture linked to poor outcomes. Our findings showed that WC outperformed BMI in identifying individuals with vertebral fractures and grade ≥2 vertebral fractures, highlighting abdominal obesity might play a more relevant role in risk of vertebral fractures than obesity. Given BMI’s dependence on both height and weight, each independently linked to fracture risk, we further assessed WHtR, a height-adjusted measure of central adiposity. Recent evidence suggests WC is rising more rapidly than BMI in multiple populations, making WHtR a more sensitive indicator of fat distribution changes (36). Notably, WHtR ≥0.5, a widely accepted threshold for central obesity, demonstrated a more superior predictive ability for vertebral fractures, and enhanced the performance of baseline models including age, sex, fracture history, and BMD. Although CVAI integrates metabolic markers, it did not provide additional predictive value beyond WC or WHtR. Moreover, ethnic differences in body composition should be considered. Compared with Western populations, Asian individuals tend to have higher body fat and visceral adiposity at a given BMI, suggesting BMI may underestimate adiposity-related skeletal risk in Chinese populations. This may partly explain why abdominal obesity indices showed stronger associations with fracture outcomes than BMI in our study (37). Overall, our results supported WHtR as an easily applicable and reliable tool for the risk of vertebral fracture assessment, offering practical clinical utility without requiring lab tests. However, abdominal obesity indices showed limited predictive power for non-spinal fractures, suggesting site-specific effects of adiposity on bone fragility.

Several mechanisms may underlie the link between abdominal obesity and increased fracture risk. Falls, a leading cause of fractures in older adults, are more frequent among individuals with abdominal obesity due to impaired physical function. In our present study, elevated WC, WHtR, and CVAI were linked to reduced balance ability. WC was linked to failure on the Romberg test, while those in the highest WHtR and CVAI quartiles had greater odds of balance impairment, which was consistent with prior research on fall-related fractures (11, 38, 39). Biomechanically, central fat shifts the center of gravity forward and upward, increasing ankle torque and compromising postural control (11). Among the indices, CVAI showed the strongest association with muscle dysfunction, likely due to its incorporation of metabolic factors. Beyond fall risk, abdominal obesity may directly impair bone quality. Visceral fat promotes chronic inflammation, which enhances bone resorption and inhibits formation, damaging bone microarchitecture and lowering trabecular bone score (40). Abdominal obesity is also associated with hormonal disturbances, including reduced GH/IGF-1 and altered sex hormones, further impairing bone remodeling (41). Additionally, increased marrow adiposity suppresses osteoblast activity (42), and vertebral structural changes may reduce spinal strength, contributing to the increased prevalence of vertebral fractures (43).

The key strength of this research is the inclusion of a sizable, population-based cohort and the novel evaluation of multiple abdominal obesity indices in relation to both fracture prevalence and balance impairment. Thorough covariate adjustment and ROC-based evaluation supported the validity of the results, certain limitations should be acknowledged. First, as a cross-sectional analysis, this study is limited to associations without causal conclusions. Second, residual confounding remains possible despite multivariable adjustments. Third, limited data on fracture site, severity, and recurrence restricted our further analysis. Fourth, only baseline abdominal obesity indices were assessed, without accounting for longitudinal changes. Fifth, anthropometric measures may not accurately reflect visceral adiposity compared to imaging-based assessments. Lastly, reliance on self-reported data for fractures, physical activity, and comorbidities may introduce recall bias and potential misclassification. In particular, fractures were not classified by trauma mechanism, and comorbidities such as hypertension and diabetes were defined based on self-report and medication use rather than formal diagnostic criteria. These factors may have introduced heterogeneity and limited the ability to isolate low-trauma fragility fractures.

## Conclusion

In summary, our study revealed that abdominal obesity was linked to higher BMD and lower bone turnover, but also impaired balance and higher prevalence of vertebral and recent clinical fractures. Among the obesity measures, WHtR showed the greatest predictive value for vertebral fractures, highlighting its potential utility in fracture risk stratification.

## Data Availability

The raw data supporting the conclusions of this article will be made available by the authors, without undue reservation.
