# Supplementary material for: Associations between abdominal obesity and the prevalence of fractures among Chinese adults: insights from a nationwide cross-sectional study
Source: Front Endocrinol (Lausanne). 2026 Feb 25;17:1759573. doi: 10.3389/fendo.2026.1759573 (PMC12975428; doi:10.3389/fendo.2026.1759573)
Supplement: Supplementary file 1 [file Table1.docx]

**Supplementary Table 1. Associations between abdominal obesity with the prevalence of fractures stratified by BMI categories**

|  | **Vertebral fractures** | | | **Vertebral fractures ≥Grade2** | | **Clinical fractures in recent 5y** | |
| --- | --- | --- | --- | --- | --- | --- | --- |
|  | **OR (95%CI)** | ***p*-value** | **OR (95%CI)** | | ***p*-value** | **OR (95%CI)** | ***p*-value** |
| **Defined by WC** |  |  |  | |  |  |  |
| NW without AO (Ref) | Ref | / | Ref | | / | Ref | / |
| NW with AO | 1.12 [0.77, 1.62] | 0.54 | 1.75 [1.06, 2.79] | | ***0.022*** | 1.00 [0.47, 1.92] | 0.99 |
| OW without AO | 1.39 [1.09, 1.75] | ***0.0071*** | 1.29 [0.86, 1.90] | | 0.20 | 1.77 [1.25, 2.47] | ***0.0011*** |
| OW with AO | 1.33 [1.08, 1.64] | ***0.0078*** | 1.63 [1.18, 2.23] | | ***0.0026*** | 1.74 [1.26, 2.39] | ***<0.001*** |
| OB without AO | 2.21 [1.06, 4.20] | ***0.022*** | 2.09 [0.61, 5.39] | | 0.17 | 1.85 [0.56, 4.60] | 0.24 |
| OB with AO | 2.03 [1.59, 2.58] | ***<0.001*** | 2.43 [1.67, 3.49] | | ***<0.001*** | 2.40 [1.70, 3.36] | ***<0.001*** |
| **Defined by WHtR** |  |  |  | |  |  |  |
| NW without AO (Ref) | Ref | / | Ref | | / | Ref | / |
| NW with AO | 1.23 [0.96, 1.58] | 0.10 | 1.42 [1.31, 2.92] | | ***0.011*** | 1.12 [0.73, 1.70] | 0.61 |
| OW without AO | 1.00 [0.53, 1.75] | 0.99 | 1.57 [0.53, 3.74] | | 0.36 | 1.64 [0.84, 2.97] | 0.12 |
| OW with AO | 1.52 [1.22, 1.89] | ***<0.001*** | 2.03 [1.40, 2.99] | | ***<0.001*** | 1.80 [1.30, 2.52] | ***<0.001*** |
| OB without AO | 1.52 [0.08, 8.59] | 0.70 | 6.51 [0.34, 39.10] | | 0.089 | 2.70 [0.15, 14.12] | 0.34 |
| OB with AO | 2.30 [1.77, 3.00] | ***<0.001*** | 3.28 [2.13, 5.09] | | ***<0.001*** | 2.36 [1.63, 3.45] | ***<0.001*** |

^1^ Abdominal obesity was defined as WC≥90cm for males or WC≥85cm for females, or participants with WHtR>0.5.

^2^ Multivariate logistic regression was used to adjust covariates compared with the Q1 group.

^3^ Adjusted for age, sex, urban-rural difference, smoking and drinking status, diabetes mellitus, hypertension, osteoarthritis, daily carbonated beverage, daily tea, daily coffee, daily exercise duration, history of fracture, the Sharpened Romberg test, and BMD at the lumbar spine.

^4^ Abbreviation: NW, normal weight group; OW, over weight group; OB, obese group; WC, waist circumference; WHtR, waist to height ratio; AO, abdominal obesity; OR, odds ratio; 95%CI, 95% confidence interval.

^5^ Significant values (p<0.05) are presented in bold italics.
